# Vascular Aging in Rodent Models: Contrasting Mechanisms Driving the Female and Male Vascular Senescence

**DOI:** 10.3389/fragi.2021.727604

**Published:** 2021-09-08

**Authors:** Paula R. Barros, Tiago J. Costa, Eliana H. Akamine, Rita C. Tostes

**Affiliations:** ^1^ Department of Pharmacology, Ribeirão Preto Medical School, University of São Paulo, Ribeirão Preto, Brazil; ^2^ Department of Pharmacology, Institute of Biomedical Science, University of São Paulo, São Paulo, Brazil

**Keywords:** vascular aging, aging, vascular dysfunction, sex difference, vascular senescence

## Abstract

Increasing scientific interest has been directed to sex as a biological and decisive factor on several diseases. Several different mechanisms orchestrate vascular function, as well as vascular dysfunction in cardiovascular and metabolic diseases in males and females. Certain vascular sex differences are present throughout life, while others are more evident before the menopause, suggesting two important and correlated drivers: genetic and hormonal factors. With the increasing life expectancy and aging population, studies on aging-related diseases and aging-related physiological changes have steeply grown and, with them, the use of aging animal models. Mouse and rat models of aging, the most studied laboratory animals in aging research, exhibit sex differences in many systems and physiological functions, as well as sex differences in the aging process and aging-associated cardiovascular changes. In the present review, we introduce the most common aging and senescence-accelerated animal models and emphasize that sex is a biological variable that should be considered in aging studies. Sex differences in the cardiovascular system, with a focus on sex differences in aging-associated vascular alterations (endothelial dysfunction, remodeling and oxidative and inflammatory processes) in these animal models are reviewed and discussed.

## Introduction

Studies involving sex differences in cardiovascular biology have increased in recent years. Many mechanisms, that differ in males and females, orchestrate vascular function and vascular abnormalities in cardiovascular and metabolic diseases. Women are hemodynamically younger than men of the same age, and cardiovascular disease is more prevalent in men than women (World Health Organization, 2015). However, after menopause, many (but not all) sex differences in cardiovascular disease are abrogated, suggesting two significant and correlated drivers: genetic and hormonal factors (World Health Organization, 2015).

With the increasing life expectancy and aging population, studies on aging-related physiological changes and aging-related diseases have steeply grown, and, with them, the use of aging animal models. Mouse and rat models of aging, the most studied laboratory animals in aging research, exhibit sex differences in many systems and physiological functions, as well as sex differences in the aging process and aging-associated cardiovascular changes. As expected, aging and senescence-accelerated animal models used to study sex differences present limitations.

**FIGURE 1:**
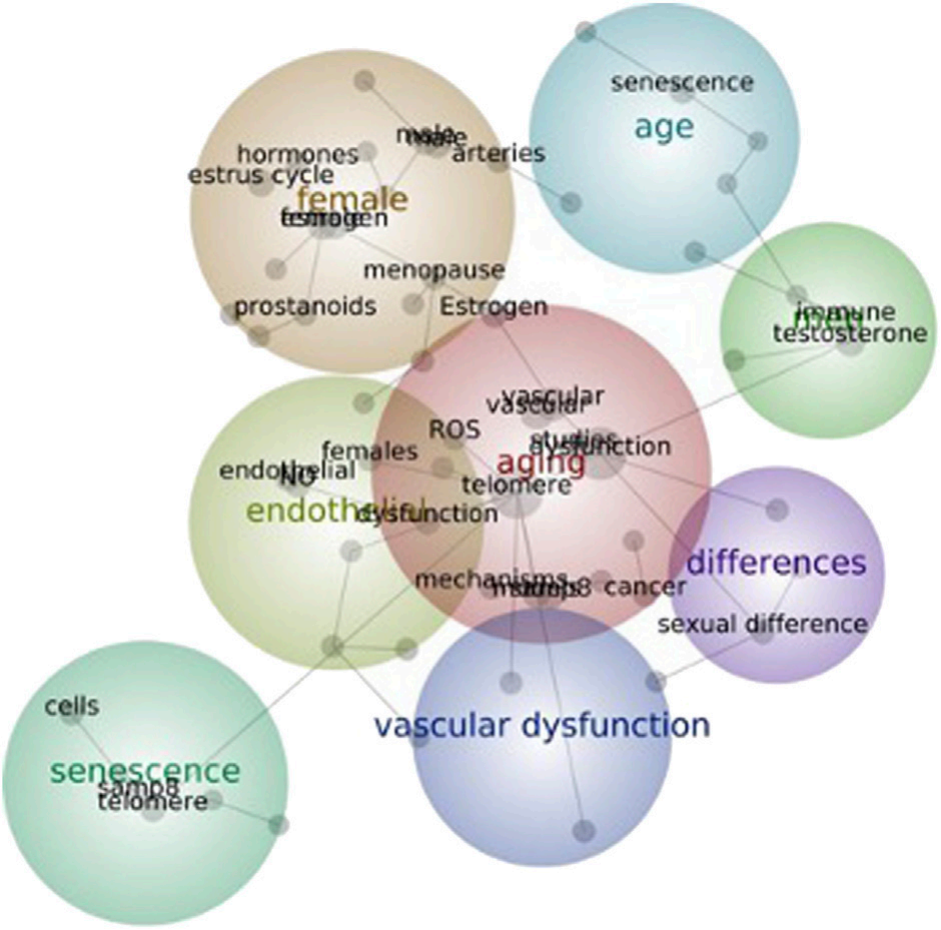
Connections within the vascular system, aging and sex themes. The Concept map was elaborated using the Leximancer algorithm, the original manuscripts and the keywords: aging, senescence, female, male, endothelium and vascular dysfunction. The lines between the concepts (grey circles) show typical pathways linking the concept terms. The size of the grey circles indicates the overall relative frequency of concepts.

Therefore, the main characteristics of aging animal models, considering sex as a biological variable are considered in the present review. Sex differences in the cardiovascular system, with a focus on sex differences in aging-associated vascular alterations (endothelial dysfunction, remodeling and oxidative and inflammatory processes) that occur in these animal models are described and discussed. Figure 1, elaborated with the Leximancer algorithm Concept, depicts a map with three primary themes (vascular dysfunction, aging and sex differences). The map reinforces the complexity and interrelationships between these variables, as indicated by the interactions between the interconnected subthemes.

### The Aging Process

Aging is an irreversible process yet little understood in human biology. Biological aging is an intrinsic, progressive, and generalized deterioration of biological homeostasis that occurs over time (Kovacic et al., 2011). It’s a complex process that involves several biological changes, in addition to the cellular senescence described by Hayflick and Moorhead (1961), as will be discussed.

López-Otín et al. (2013) identified and categorized the cellular and molecular characteristics of aging, proposing a total of nine markers that, together, can determine the aging phenotype: 1) genomic instability, 2) shortening of telomeres, 3) epigenetic changes, 4) loss of proteostasis, 5) unregulated detection of nutrients, 6) mitochondrial dysfunction, 7) cell senescence, 8) stem cell exhaustion, and 9) altered intercellular communication. These processes are interrelated, suggesting that aging is not the result of the malfunction of a single parameter, but rather a combination of different molecular changes. With the progression of aging, the changes in these many processes coincide with the emergence of age-related diseases that alter the body homeostasis and reduce the quality of life (Da Costa et al., 2016; Lees et al., 2016).

In 2050, 1 in 6 people in the world will be over the age of 65, up from 1 in 11 in 2019 (United Nations, 2019). Older people account for more than one fifth of the population in 17 countries and in 2,100 this will be the case for 155 countries, covering 61% of the world’s population (United Nations, 2019). These numbers represent a public health problem, since aging remains the strongest risk factor for cardiovascular disease (CVD) (World Health Organization, 2015), even after correction for classic cardiovascular risk factors, such as smoking, physical inactivity, arterial hypertension, diabetes, and obesity (Lakatta and Levy, 2003; Erusalimsky, 2009; Erusalimsky and Skene, 2009).

### Sex Differences in Aging

Physiological mechanisms that control vascular function are different in men and women. Mechanisms involved in diseases-associated vascular dysfunction also seem to follow a different pattern of development in males and females. In aging, these sex differences are already seen in life expectancy, where women currently outlive men by 4.8 years (United Nations, 2019).

Sex hormones are responsible for the most marked endocrine changes with aging (Horstman et al., 2012). In men, aging-related changes, including sexual dysfunction, decreased muscle strength, muscle and joint pain, insomnia, and physical exhaustion (Heinemann, 2005), usually appear as early as in middle age, 40- to 59-year-old men, with reduced testosterone levels since the age of 35–40 (Feldman et al., 2002). In women, aging is associated with the postmenopausal period, linked to a decrease in the natural sex hormones, estrogen and progesterone, and increased follicle-stimulating hormone (FSH) levels (El Khoudary et al., 2020). Although the risk for CVD increases with the aging process in both sexes, it is more severe in postmenopausal women (El Khoudary et al., 2020). The mechanisms that determine the aging condition in men and women are targeted by a growing number of studies (Nakamura and Miyao, 2008; Austad, 2019).

The aging of the population will cause an increasing burden to the health systems, implying profound changes in public health policies. Therefore, it is very important to improve our knowledge on the changes that occur in biological systems during men and women aging, since this might reveal potential therapeutic targets to reduce aging-related cardiovascular dysfunction, and prevent lethal or debilitating cardiovascular events.

### Cellular Senescence

Hayflick and Moorhead (1961) introduced the term “senescence” to describe the phenomenon of irreversible growth blockade of human diploid cells in culture after extensive series passages, later known as “replicative senescence”. At some point, cells arrive at a steady state, called the Hayflick limit, and they become senescent (Hayflick and Moorhead, 1961). Long after the Hayflick and Moorhead discovery, and considering Olovnikov’s end-replication telomere loss problem (Olovnikov, 1971; Olovnikov, 1973), the molecular mechanism underlying the Hayflick limit was explained: the shortening of telomeres. Telomeres are repeated sequences, rich in T-G bases (Xu et al., 2013) sequenced as 5′TTAGGG3′ in vertebrates (Hemann and Greider, 1999) found at chromosome ends, which shorten with each cell duplication. The telomeres shortening or the loss of telomere t-loop structure (Olovnikov, 1973; Levy et al., 1992; Greider, 2000) is sensed by the cell as a DNA damage, and cell signaling is activated within to stop the cell cycle and to increase a pro-inflammatory secretory phenotype (Van Deursen, 2014), supporting the original hypothesis of Hayflick and Moorhead, in which senescence protects against the unrestricted growth of damaged cells. Subsequently, it was found that not only the cells of an elderly individual become senescent, but that all differentiated cells, such as fibroblasts, have a limited division potential before undergoing the so-called “replicative senescence”.

Cellular senescence can be seen as an important physiologic mechanism of protection. Unlike a static endpoint, senescence represents a very dynamic cellular process that can happen in different states of the cell to protect against different cell stressors, as seen in autophagy and cancer (Slobodnyuk et al., 2019), embryonic development (Rajagopalan and Long, 2012; Muñoz-Espín et al., 2013; Storer et al., 2013), tissue repair (Krizhanovsky et al., 2008; Jun and Lau, 2010), aging and age-related disorders (Baker et al., 2008). This leads to a couple of questions: how can senescent cells be present in such antagonic situations as embryonic development and aging? Do they have different roles? Are there different types of senescence in cells? A recent review Van Deursen (2014) discussed that senescent cells can be divided into two types: acute and chronic senescent cells, based on the kinetics of senescence induction and functionality.

Acute senescent cells are part of a tightly orchestrated biological processes (that is, wound healing, tissue repair, embryonic development) induced through cell-extrinsic stimuli that target a specific population of cells in the tissue. This process aims to halt expansion of certain cells or to produce a senescence-associated secretory phenotype (SASP) with well-defined paracrine functions. For example, in wound closure or tissue development, myofibroblasts suddenly undergo senescence (acute senescence) to limit excessive fibrosis at the site of injury (Jun and Lau, 2010) such as in liver damage (Krizhanovsky et al., 2008). Acute senescent cells self-organize by releasing SASPs to attract immune cells in charge for their resolution/elimination in the tissue (Van Deursen, 2014).

Chronic senescent cells arise after periods of progressive cellular stress and chronic senescence does not seem to target specific cell types. Possibly due to age-related immunodeficiency or decreased production of pro-inflammatory SASPs, as exemplified by melanocytic nevi (Benz et al., 1991), immune cells may inefficiently eliminate chronic senescent cells, allowing continuation of multi-step senescence. In addition, in aging-related senescence, the switch from temporal to persistent cell-cycle arrest appears unscheduled, probably involving the combined effects of distinct senescence-inducing stressors acting simultaneously on a cell (Van Deursen, 2014).

But can acute and chronic senescent cells permeate between those states? Senescence induced during cancer therapy may initially be acute and later chronic in nature (Roberson et al., 2005; Gewirtz et al., 2008), but the other way around is still controversial in the literature. It is possible that senescence may initially have arisen as a developmental or wound healing mechanism that has only recently in evolutionary time adapted as a tumor suppressor mechanism with aging as a side-effect (Campisi, 1997). Cultured cells usually reach senescence within several weeks after exposure to senescence-inducing stressors but remain viable for months thereafter (De Cecco et al., 2013). Senescent cells continue to evolve even after extended periods of culture, thereby progressing to a stage that has been termed “deep” or “late” senescence.

The exact point where senescent cells fail to manifest their beneficial effects through the SASP and promote a tissue-specific disease is still unknown. So, at what time would it be desirable to eliminate the senescent cells? This evidence implies that senescence and other cellular responses to stress might be related to life span and health span, not only the lack of them, but also an overstated response, and might provoke disease. Not all cells become senescent at the same time, but the senescent phenotype can be transmitted to neighboring cells (Hoare and Narita, 2013). So, what if the senescent cells that perform the beneficial effects are not the first ones that became senescent? It might be necessary the accumulation of a certain number of senescent cells to carry out these effects; and what if those cells were eliminated before they could induce senescence in their neighbor cells? These questions still need to be answered in aging research.

#### Sex Differences in Senescence

Males show higher senescence rates than females and this sex difference is largely attributable to sex-specific downstream effects of the intensity of intra-sexual competition experienced during early adulthood (Promislow, 1992; Maklakov and Lummaa, 2013; Chen and Maklakov, 2014). However, one of the criticisms is that these studies are carried out in species with low extrinsic mortality and the mechanisms that generate such differences remain poorly understood (Beirne et al., 2015).

Since senescence has been related to telomere shortening, the influence of sex on telomere suggests that a sex bias in telomere maintenance does exist, since female rats might have greater telomerase activity (Leri et al., 2000). In fact, estrogen activates a promoter of telomerase (Kyo et al., 1999) and indirectly also affects DNA repair through the p53 pathway. An estrogen-responsive element (ERE) is present in telomerase reverse transcriptase (hTERT) indicating that estrogen might stimulate telomerase to add telomere repeats to the ends of chromosomes (Nordfjäll et al., 2005). Telomeres are particularly sensitive to oxidative stress (Van Der Harst et al., 2007) and, in physiological conditions, the levels of reactive oxygen species (ROS) are lower in the vascular system of premenopausal women than men (Ide et al., 2002; Kander et al., 2017). A meta-analysis study suggested that women have longer telomeres than men irrespective of cell type or age. It’s important to mention that different techniques are used to measure telomeres length, such as real-time PCR, Flow-FISH, and Southern blotting. However, only Southern blotting showed significant differences in mean telomere length between the sexes (Gardner et al., 2014), while other studies showed that women does not always have longer telomeres (Hunt et al., 2008; Shiels et al., 2011).

Endothelial senescence has been more and more explored, especially because aging *per se* is a risk factor for endothelial dysfunction (Erusalimsky, 2009; Erusalimsky and Skene, 2009). In the vascular system the problem is that *in vitro* studies of cellular endothelial senescence have traditionally been performed using a single senescence-inducing stimulus in endothelial cells: mitogens (Kurz et al., 2003), inflammatory molecules (Breitschopf et al., 2001) or ROS (Kurz et al., 2004). However, in the context of organismal aging, individual cells experience multiple cellular pressures, including various kinds of genotoxic, proteotoxic and mitotic stresses (Hayflick and Moorhead, 1961; Siegel and Amon, 2012). Thus, to advance our understanding of these processes, one should examine how combinations of diverse senescence-promoting stressors impact the actions of the various downstream effector pathways and whether the characteristics of the resulting SASP vary in distinct cell types and under different senescence-inducing stressors (Coppé et al., 2008). The SASP produced by senescent cells might be a good parameter to explore the effects of several senescence-inducing stimuli in endothelial cells from males and females. There are several SASPs that depend on persistent DNA damage signaling (Rodier et al., 2009) and that is independent of DNA damage (Freund et al., 2011), implying the existence of DNA damage response-independent mechanisms (Kaplon et al., 2013; Muñoz-Espín et al., 2013; Storer et al., 2013).

## Experimental Animal Models for Aging Studies

### Models to Study Aging

According to the National Institutes of Health (National Human Genome Research Institute), an animal model is a non-human species used in medical research because it can mimic aspects of a disease found in humans. Animal models are used to obtain information about a disease and its prevention, diagnosis, and treatment. By using animals, researchers can carry out experiments that would be impractical or ethically prohibited with humans (sic) (National Human Genome Research Institute, 2021).

In that context, a few common model organisms are studied in aging research, such as single-cell yeast, *Saccharomyces cerevisiae*; the nematode *Caenorhabditis elegans*; the fruit fly *Drosophila melanogaster*, the laboratory mouse (*Mus musculus*) and rat (*Rattus norvegicus*). These species span a considerable distance in animal evolution, but shared features of these evolutionary divergent animals strongly indicate the presence of some conserved processes in aging (Mitchell et al., 2015). However, there is still considerable debate concerning the extent of generality of ageing mechanisms in experimental animal models.

For several years, the use of invertebrate animal models such as *Caenorhabditis elegans* or *Drosophila melanogaster*, has led aging research by providing the first insights into those molecular pathways that are determinant in the aging process and for lifespan extension. However, for vascular aging studies, mouse models - in comparison with other mammals - are more complex, faster, and cheaper tools for lab research. Specifically, inbred aged mice models, such as the C57BL/6J mice, are commonly used in ageing vascular studies (Flurkey et al., 2007).

### Rodent Models to Study Natural Aging

As shown in a large cohort study with C57BL/6J mice (150 males and 150 females), the maturational rate of mice does not linearly correlate with humans. It occurs 150 times faster during the first month of life and 45 times faster over the next 5 months, during which mice pass through their mature adult stage. Mature adult mice, age from 3–6 months, are often used as the reference group (control) in aging studies, since this group is past development but not yet affected by aging outcomes. Although sexual maturity is reached around 35 days, rapid growth continues until about 3 months, with the life phase equivalent for humans ranging from 20–30 years (Flurkey et al., 2007). Middle-aged groups help determine if an age-related change is progressive or is first expressed only in old animals. Mice should be at least 10 months-old and up to 15 months-old for inclusion in a middle age group. This phase correlates to humans that are 38–47 years-old. Mice at age 18–24 months correlate with humans in their 56–69 years. This age range meets the definition of “old,” which is the presence of changes in almost all biomarkers of senescence. It is important to carefully choose the age cohorts such that animals used are neither too young nor too old. Animals that are nearing the end of their lives may already be riddled with age-associated diseases, whereas those that are too young may still be undergoing the complex process of development and maturation (Mitchell et al., 2015).

Regardless of age, animal models should be healthy, pathogen- and disease-free, and have no signs of tumors or lesions (Storer, 1966). The use of necropsy data, the dangers of pooling samples from different individuals, planning ahead for loss of aged mice to death and disease, the use of cost-adjusted power calculations, and the dangers of inferring causal associations from correlated age effects are important issues to be considered in aging research (Miller and Nadon, 2000).

Another important issue is that aging studies should be undertaken in both genders, for differences in genotype may be evident. For example, old male inbred CBA/J mice have a stronger likelihood of developing hepatocellular tumors than females (Nadon, 2006). Similarly, quantitative trait locus mapping of Drosophila genes has identified gender specific loci that differentially alter longevity (NuzhdinMackay and Mackay, 1994).

In terms of female vascular aging, a study in a model that also presents the menstrual phase of women would be ideal, but few species experience menstruation and this type of research is oftentimes ethically difficult and costly to perform. Although rats do not experience menses, they do experience estrus cycling and ovarian aging. Reproductive maturity of rodents is reached at 3–5 months, when there is an estrous cycle that lasts four to 5 days. Similarly, as women age, there is a progressive gradual decline in estrogen levels caused by changes in the hypothalamic–pituitary control of gonadotropin secretion and gonadal stimulation of estrogen (Yuan et al., 2005). Aged female rats and mice exhibit periods of persistent estrous cycle, consisting of elevated and constant levels of estradiol, low levels of progesterone, and lack of luteinizing hormone (LH), in addition to ovulation (Lu et al., 1979; Simpkins et al., 1979; Yuan et al., 2005). Ovarian function declines between 3 and 6 months in the senescence-accelerated mouse prone 8 (SAMP8) model (NuzhdinMackay and Mackay, 1994) and at 10–12 months in Long Evans rats (Lu et al., 1979). Therefore, reproductive maturity depends on the rodent strain and is characterized by low levels of estradiol and progesterone, with little or no developing follicles and increased prolactin secretion (Advis et al., 1978; Lu et al., 1979; Simpkins et al., 1979).

### Senescence-Accelerated Mouse Models

#### Genetically-Engineered Mouse Models

The disadvantages of working with vascular aging in animals include 1) the long waiting time for the animal to grow old, 2) the long duration of the studies and 3) the different ages at which the studies are performed (Folkow and Svanborg, 1993; Küng and Lüscher, 1995; Zhou and Frohlich, 2003; Zieman et al., 2001). The main reason is undoubtedly the financial burden: aged mice must be either purchased (e.g., $106 to grow a mouse for 2 years) or “matured” from a young age to 18–24 months, also expensive and time-consuming (Miller and Nadon, 2000).

Mice with accelerated vascular aging provide an alternative that saves time and energy. Mice with progeroid syndromes [well described in Liao and Kennedy (2014)] are widely used in aging research. These mice are termed progeroid, which means resembling premature aging, which in some cases involve mutations in the same genes that have been linked to human progeria syndromes (Burtner and Kennedy, 2010; Cox and Faragher, 2007; Kudlow et al., 2007). Most progeroid syndrome animal models are developed with a single gene deletion that leads to a strong phenotypic overlap with normal aging lesions (Table 1), well discussed by two reviews (Mitchell et al., 2015; Kõks et al., 2016).

**TABLE 1:** Genetically-engineered mouse models regularly used in aging research and the age-distinguishing characteristic (hallmark) in males and females.

Mouse model	Gene targeting	Human syndrome	Age hallmark	References
Ercc1 ^-/-^	Ercc1 knockout	XFE progeroid syndrome	Genomic instability	Weeda et al. (1997)
Ercc1^-/Δ7^	Ercc1 hypomorphic		Genomic instability	Dollé et al. (2011)
Ercc^2R722W/R722W^, Xpd^TTD/TTD^	Ercc2 knockin	Trichothiodystrophy	Genomic instability	de Boer et al. (2002)
Ercc4^m/m^, Xpf^m/m^	Ercc4 knockout	Xeroderma pigmentosum group F	Genomic instability	Tian et al. (2004)
Ercc5^−/−^, Xpg^−/−^	Ercc5 knockout	Xeroderma pigmentosum group G/Cockayne syndrome	Genomic instability	Barnhoorn et al. (2014)
Ercc6^m/m/^Xpa^−/−^, Csb^m/m^/Xpa^−/−^	Double Ercc6/Xpa knockout	Cockayne syndrome	Genomic instability	van der Pluijm et al. (2007)
Xrcc5^−/−^, Ku80^−/−^ or Ku86^−/−^	Xrcc5 knockout		Genomic instability	Li et al. (2007)
Xrcc6^−/−^, Ku70^−/−^	Xrcc6 knockout		Genomic instability	Espejel et al. (2004)
Prkdc^−/−^, Xrcc7^−/−^ or DNA-PKcs^−/−^	Prkdc knockout		Genomic instability	Li et al. (2007)
Wrn^−/−^/Terc^−/−^	Double Wrn/Terc knockout	Werner syndrome	Genomic instability	Espejel et al. (2004)
Bub1b^H/H^, BubR1^H/H^	Bub1b hypomorphic		Genomic instability	Chang et al. (2004)
Bub1b^+/GTTA^, BubR1^+/GTTA^	Bub1b knockin	Mosaic variegated aneuploidy syndrome	Genomic instability	Baker et al. (2004)
Bub3^+/−^/Rae1^+/−^	Double Bub3/Rae1 haploinsufficient		Genomic instability	Wijshake et al. (2012)
SprtnH/H	Sprtn hypomorphic	Ruijs-Aalfs syndrome	Genomic instability	Baker et al. (2006)
Arhgap1^−/−^, Cdc42GAP^−/−^	Arhgap1 knockout		Genomic instability	Maskey et al. (2014)
Atr^S/S^	Atr hypomorphic	Seckel syndrome	Genomic instability	Wang et al. (2007)
Atr^flox/−^:Cre-ERT2^+^	Atr inducible knockout	Seckel syndrome	Genomic instability	Murga et al. (2009)
Polg^D257A/D257A^, mtDNA mutator mouse	Polg knockin		Genomic instability	Ruzankina et al. (2007)
Lmna^G609G/G609G^, LAKI mouse, Lmna^L530P/L530P^, Lmna^HG/+^, Lmna^H222P/H222P^	Lmna knockin	Hutchinson-Gilford progeria syndrome	Genomic instability	Osorio et al. (2011); Trifunovic et al. (2004); Mounkes et al. (2003)
Zmpste24^−/−^	Zmpste24 knockout	Hutchinson-Gilford progeria syndrome	Genomic instability	Bergo et al. (2002); Pendás et al. (2002)
Terc^−/−^	Terc knockout	Dyskeratosis congenita	Telomere attrition	Rudolph et al. (1999)
Tert^ER^	Tert knockin	Dyskeratosis congenita	Telomere attrition	Jaskelioff et al. (2011)
Tert^−/−^	Tert knockout	Dyskeratosis congenita	Telomere attrition	Bär et al. (2016)
Sirt6^−/−^ and Sirt1^-/-^	Sirt1-6 knockout		Epigenetic alterations	Mostoslavsky et al. (2006); Mercken et al. (2014)
Bmi1^−/−^	Bmi1 knockout		Epigenetic alterations	van der Lugt et al. (1994)
Sod1^-/-^ and Sod2^-/-^	Sod1-2 knockout		Oxidative stress	Li et al. (1995); Elchuri et al. (2005)
MsrA^-/-^	MsrA gene knockout		Oxidative stress	Moskovitz et al. (2001)
Prdx1^-/-^	Prdx1 gene knockout		Oxidative stress	Neumann et al. (2003)
Kl^kl/kl^, Klotho^kl/kl^	Kl knockout		Altered intercellular communication	Kuro-o et al. (1997)
bGH-Tg, GH-transgenic mice	Overexpression of Growth Hormone		Somatotropic (GH/IGF-1) axis	Casellas and Medrano, (2008)
Nfkb1^−/−^	Nfkb1 knockout		Altered intercellular communication	Bernal et al. (2014)
Il10^tm/tm^, Frail mouse	Il10 knockout		Altered intercellular communication	Kühn et al. (1993); Walston et al. (2008)

HGPS, Hutchinson-Gilford progeria syndrome; ERCC1, excision repair cross complementing 1; IL-10, interleukin-10; Lmna, Lamin A; PolG, Polymerase γ; Terc, Telomerase RNA component; Wfs, Wolfram syndrome; Wrn, Werner syndrome ATP-dependent helicase; WS, Werner syndrome; XPD, xeroderma pigmentosum, complementation group F; Zmpste24, zinc metalloproteinase Ste24; CHIP, carboxyl terminus of Hsp70-interacting protein; MsrA, Methionine sulfoxide reductase; Prdx1, peroxiredoxin 1.

Although transgenic mice have been proposed as an useful approach to study aging (Musarò and Rosenthal, 1999; Warner and Sierra, 2003), this multifactorial phenomenon will hardly be simulated by monogenic approaches. It also remains highly debatable to what extent the molecular events leading to progeria overlap with those driving normal aging (Hasty and Vijg, 2004; Kipling et al., 2004; Miller, 2004). Biogerontologists are increasingly realizing that “single molecule, single target” approaches for aging interventions are severely limited due to the highly dynamic, interactive and networking nature of life. In addition, this approach limits the number of variables under study, and usually ignores synergistic interactions, thereby oversimplifying the process.

#### Non Genetically-Modified Mouse Models

In the group of nongenetically-modified animals, the inbred strain with accelerated senescence SAMP is considered a useful animal model to study aging-related processes (Takeda et al., 1981). The SAMP model allows a shorter waiting time for the animal to grow old and shorter duration of the studies, compared to commonly used old rats and other mouse inbred strains (Goodrick, 1975; Flurkey et al., 2007) (Table 2).

**TABLE 2:** Non genetically-modified mouse models regularly used in aging research and their relative ages of study in males and females.

Animal models	Maturation (puberty)	Young (sexual maturity)	Middle-aged (fall of reproductive functions)	Aged	Life-span (average)	References
C57BL6	±28 days	3–6 months	10–15 months	18–24 months	2 years	Dutta and Sengupta, (2016); Yuan et al. (2009)
Wistar rat	±28 days	5–6 months	≥18 months	≥24 months	3 years	Sengupta, (2013)
Fischer 344 rats	±28 days	4–6 months	≥18 months	≥24 months	1.75 years	Chesky and Rockstein, (1976)
SAMP8	±28 days	2–3 months	≥6 months	≥8 months	12.1 months	Takeda et al. (1981); Takeda, (1999)
SAMR1	±28 days	2–3 months	≥8 months	≥10–15 months	18.9 months	Takeda et al. (1981); Takeda, (1999)
CD1 mice	±22 days	3 months	≥8 months	≥12–18 months	2 years	Eveleigh et al. (1983); Russell and Green, (2007)

Currently, there are nine SAMP strains (SAMP- 1, 2, 3, 6, 7, 8, 9, 10, and 11) and three SAMR (senescence-accelerated resistant mouse prone) inbred strains (SAMR-1,4, and 5) (Takeda, 1999). The strains were characterized according to certain common characteristics. The strain SAMP-8, SAMP-6 and SAMP-10 were widely used as a model of age-related disease, such as cognitive deficit, due to spontaneous accumulation of amyloid beta plaques similar to Alzheimer’s disease, cardiac dysfunction and dysregulation of the immune system (Butterfield and Poon, 2005; Forman et al., 2011). On the other hand, other SAMP strains were reported to present senile osteoporosis (SAMP6); contracted kidney (SAMP1, SAMP11); impaired immune response, hyperinflation of lungs, hearing impairment and hypertensive vascular disease (SAMP1); degenerative temporomandibular joint disease (SAMP3); thymic lymphoblastic lymphoma and abnormal circadian rhythms (SAMP9, SAMP7); cataracts (SAMP9) and brain atrophy (SAMP10) (Takeda, 1999).

Although it should be kept in mind that inbred strains are limited in genetic diversity and, hence, develop phenotypes specific to those strains, they are easy to genetically manipulate and extensive baseline data are available (Goodrick, 1975). On the other hand, outbred mice are more representative of the genetic diversity of humans, but they present alterations in the genome (Kõks et al., 2016).

Many mouse models to study mechanisms of aging have been developed. With an increasing world older population, those models will be extremely important to test aiming to improve future interventions on aging-related diseases. Although mouse models have not always been generally accepted to study the complexity of aging, much of the progress in this field can be attributed to them (Hasty et al., 2003; Burtner and Kennedy, 2010; Kennedy et al., 2014). So far, there are no gold standard markers that classify aging in animals, but the more closely the model resembles the disease situation, the more relevant will be the data generated from them. Here we will highlight some studies in vascular aging featuring males and females, with a spotlight in vascular studies in female and male SAMP mice.

## Sex Differences in Aging-Associated CVD

### Cardiovascular Aging Characterization: What is that?

During the past 2 decades, the sustained efforts to characterize the effects of aging on multiple aspects of cardiovascular structure and function and the dominant aspects of vascular aging have been reported in two major clinical studies: The Framingham cohort study (Framingham Heart Study - FHS) and the Baltimore Longitudinal Study on Aging (BLSA) (Lakatta and Levy, 2003; Alghatrif et al., 2017).

Aging-linked changes are well evident and studied in elastic arteries, such as the aorta and its main branches. In general, aging is associated with the thickening of the wall in large elastic arteries. With the thickness of the intima-media layer of carotid arteries increasing 2–3 times between the 20th and the 90th year of age (Lakatta, 1993; Lakatta and Levy, 2003). The evidence of age-associated arterial mechanical alterations is observed by the third decade of age with sharp declines in aortic strain. Aortic distensibility is the most sensitive marker of aortic aging in individuals ≤50 years of age, beyond the influences of gender, body size, and cardiovascular risk factors (Redheuil et al., 2010).

Nearly 80% of the total decline of aortic strain occurs before the fifth decade of age and is associated with an exponential increase in femoral-carotid pulse wave velocity as reported in a study with 54 men/57 women, average age of 20–84 years (Redheuil et al., 2010). Preliminary analysis from the BLSA shows that, even though women have a greater increase in pulse pressure with aging, pulse wave velocity is higher in men, due to a more accelerated increase in aortic root diameter in men than women (Lakatta, 2018). However, the observation that pulse wave velocity, which is a good measure of aortic wall stiffness, remains comparable or lower in older women as compared to men, suggests that factors other than aortic wall stiffness may contribute to the higher pulse pressure in women (Alghatrif et al., 2013). The pulse wave velocity is also an independent predictor of the future increases in systolic blood pressure and of incident hypertension (Najjar et al., 2008). An increased pulse wave velocity reflects 3 potential risk factors: increased systolic pressure, widened pulse pressure, and altered vascular wall properties.

The thickness of the vascular wall is related to the composition of the vascular wall (the amount of smooth muscle, elastin fibers and collagen) and the blood pressure that the vessel will be submitted to. Indeed, aging is the strongest predictor of arterial stiffness (Vlachopoulos et al., 2010). *Post-mortem* studies indicated that the aging-related thickening of the aortic wall consists mainly of thickening of the intima layer, even in populations with a low incidence of atherosclerosis (Virmani et al., 1991), being characterized by increased collagen deposition and the presence of disorganized smooth muscle cells (Lakatta et al., 2009).

Past 50 years of age, as central arterial strain becomes reduced, there is an early increase in diastolic blood pressure and mean arterial pressure in both sexes (Franklin et al., 1997; Scuteri et al., 2014). Indeed, diastolic blood pressure is a stronger predictor of coronary heart disease risk than systolic blood pressure or pulse pressure in ≤50 years old adults. With advancing age, as central arteries stiffen, there is a gradual shift from diastolic blood pressure to systolic blood pressure and eventually to pulse pressure as predictors of coronary heart disease risk in elderly (Franklin et al., 2001), with pulse pressure specifically in elderly women. A sex specific combination of aortic stiffening and aortic dilatation in women may account for the monotonic rates at which systolic and diastolic BP change and pulse pressure increases in women, but not in men (Scuteri et al., 2014).

Male SAMP8 (Reed et al., 2011) and female SAMP8 mice fed a western-type diet (Gevaert et al., 2017) have isolated early age-dependent diastolic dysfunction related to heart fibrosis in the absence of alterations in systolic function and blood pressure (Reed et al., 2011; Gevaert et al., 2017). SAMP8 male mice exhibit no alterations in arterial elastance, the ratio of ventricular-vascular coupling or impaired cardiac myocyte relaxation, indicating that the diastolic dysfunction cannot be explained by increased vascular stiffness or abnormalities in the interaction between the heart and the systemic vasculature. So, perhaps this might represent a good model to study mechanisms of aging-related diastolic dysfunction in males.

The aging heart is subjected to an increasing systolic load imposed by stiffening of the vasculature, a stimulus for left ventricular hypertrophy (Lakatta, 1994). Data from the FHS with 142 subjects (63 men/79 women, mean age 57 ± 9 years) and the BLSA study with 336 subjects (136 men/200 women, mean age 56 ± 18 years) show different perspectives for left ventricular mass with age. In the FHS study left ventricular mass and wall thickness were all greater in men than in women, regardless of adjustment for height or body surface area. Also, there were no changes with age in cavity dimensions in the short-axis plane in either gender (Salton et al., 2002). In the BLSA study, left ventricular long-axis length index, another independent morphometric determinant of left ventricular mass, decreased by 9.2% through adulthood, but wall thickness increased, resulting in an altered shape, but no change in overall left ventricular mass in women (Hees et al., 2002). Even though systolic load increases between the ages of 50–60 years in both sexes, left ventricular wall thickness seems more important in aging women than men, and cellular necrosis or apoptosis are considered potent players in age-related left ventricular remodeling in men (Olivetti et al., 1995; Hees et al., 2002).

The incidence CVD is lower in premenopausal women compared with age-matched men, yet menopause women surpassed that of men (Virani et al., 2020). Premenopausal women have lower autonomic tone and baroreceptor response as well as better overall vascular function than men of similar age (Barnett et al., 1999; Christou et al., 2005). Postmenopausal women have stiffer arteries than their male counterparts (Mitchell et al., 2008) even after correcting for body size and aortic diameter (Mitchell et al., 2004). Potentially related to declines in ovarian function and estrogen levels, these vascular differences are clinically reflected in patterns of hypertension prevalence over the life course. Indeed, prior to the age of 45 years, more men than women have hypertension; between 45 and 64, hypertension rates are similar between the sexes, and at ages >65 years, more women than men are hypertensive (National Center for Health Statistics, 2015). This emphasizes the need for age/gender specific reference values, and use of gender-different threshold values for cardiovascular medical exams. According to Novella and collaborators (Novella et al., 2012a), the onset of menopause overlaps with aging-associated changes, making it particularly difficult to distinguish between the contributions of aging and the lack of estrogen to vascular damage. Additional studies using animal models of ovariectomy (OVX) and data collected from ovariectomized women in clinical trials will help to clarify this point.

**FIGURE 2:**
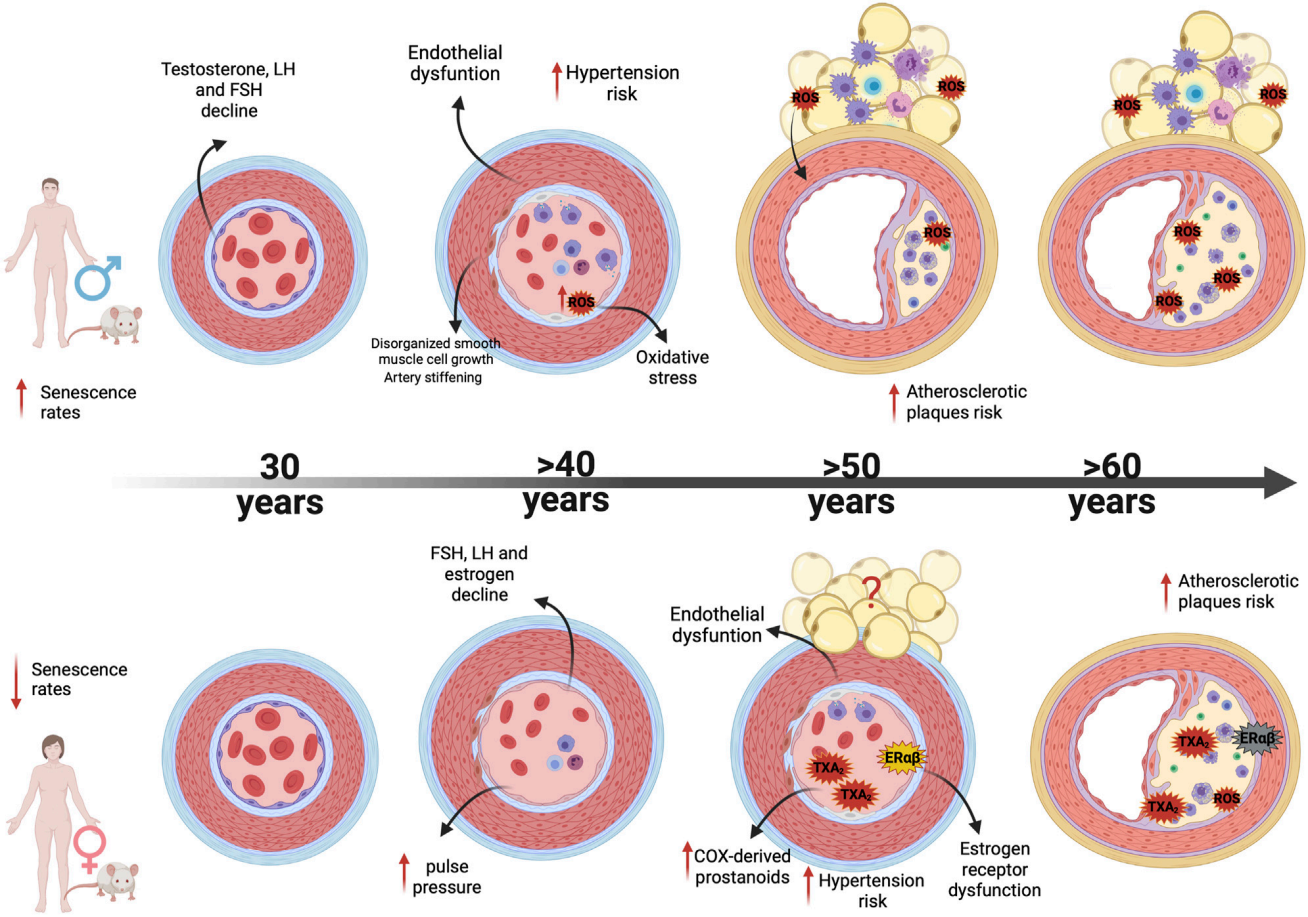
Mechanisms associated with sex differences in vascular aging. Figure illustrates vascular and PVAT age-associated alterations observed in male and female; from differences in age onset of vascular dysfunction (males show higher senescence rates than females) to different mechanisms of endothelial dysfunction, vascular remodeling and oxidative and inflammatory processes as men and women age increases. Abbreviations: LH, Luteinizing Hormone; FSH, Follicle-stimulating hormone; TXA2, Thromboxane; ERα, Estrogen receptor alpha; ERβ, Estrogen receptor; ROS, Reactive Oxygen Species.

Among the potential mechanisms involved in cardiovascular aging, endothelial dysfunction is central, since arterial remodeling in healthy humans occurs in the context of age-associated endothelial dysfunction (Celermajer et al., 1994), which is also one of the main processes by which aging increases the risk of CVD in both sexes (Celermajer et al., 1994; Gerhard et al., 1996; Taddei et al., 1997; Rodríguez-Mañas et al., 2009) as summed up in Figure 2. Endothelial dysfunction is clearly multifactorial and aging-induced endothelial dysfunction results from an imbalance characterized by increased production of ROS, increased cyclooxygenase (COX)-derived vasoconstrictor factors, and reduced bioavailability of endothelium-derived nitric oxide (NO) (El Assar et al., 2012). But what are exactly the contributions of these mechanisms for endothelial dysfunction in both sexes?

## Possible Mechanisms Whereby Sex Differences Impact the Vascular Aging Process

### The Endothelium

Age-related endothelial dysfunction is common to most arteries independently of either vascular bed and species studied (Küng and Lüscher, 1995; Novella et al., 2010; Novella et al., 2013a; Hongo et al., 1988; Haidet et al., 1995; Taddei et al., 1995; Egashira et al., 1993). Decreased endothelium-dependent vasodilation (EDV) is one of the most studied effects of aging in the vascular system. In clinical studies the rate of decline of EDV is different between aged men and women. Men demonstrate a gradual decline after the fourth decade, while women’s decline is delayed approximately one decade, but accelerated after menopause (Celermajer et al., 1994; Taddei et al., 1996). Aging is associated with a progressive decline in EDV in large conduit arteries (Lieberman et al., 1994; Lieberman et al., 1996) and peripheral resistance vessels (Delp et al., 1995; Higashi et al., 2006; Lloréns et al., 2007; Novella et al., 2013b; Jiménez-Altayó et al., 2013; Costa et al., 2019) in healthy adults. Estrogen treatment (1 mg dose for 9 weeks) in postmenopausal women at a relatively young age (average age 55 ± 7 years) improved flow-mediated endothelium-dependent dilation (Lieberman et al., 1994; Lieberman et al., 1996). In men, the reduced EDV was improved after infusion of the NO synthase co-factor, tetrahydrobiopterin (BH4) (Higashi et al., 2006).

To understand the mechanisms behind the reduction of EDV in female vascular senescence in mice, researchers have used the SAMP-8 mouse model. SAMP8 mice were used to characterize female vascular aging with and without the deleterious effects of estrogen withdrawal. Aortas of 6- to 7-month-old female SAMP-8 mice develop increased contractility and endothelial dysfunction, which is mediated by decreased NO production (Novella et al., 2010; Novella et al., 2013a). However, mesenteric arteries from the same 6- to 7-month-old female SAMP-8 mice show no decrease in endothelium-dependent vasodilation (Jiménez-Altayó et al., 2013), indicating that the functional vascular alterations in female SAMP-8 occur earlier in conductance arteries than in resistance arteries.

The increased vasoconstrictor responses, endothelial dysfunction, and reduced NO production in aorta of female SAMP-8 mice are potentiated by the lack of estrogen, i.e., by OVX performed at 5 months of age. Accordingly, a 4-weeks treatment with estrogen, started immediately after OVX, improved vascular function (Novella et al., 2013a). Of importance and aligned with clinical data, Costa et al. (2019) demonstrated that estrogen treatment loses its beneficial vascular actions in 6-month-old ovariectomized female SAMP-8 mice, either when administered shortly after OVX or 45 days after OVX, suggesting an important interaction between aging and the onset of estrogen treatment for the vascular function.

Furthermore, vasodilation to acetylcholine is completely inhibited by L-NG-Nitroarginine Methyl Ester (L-NAME) in female SAMP8 mice at the age of 3, 6 and 8 months (Novella et al., 2013b), reinforcing that changes in NO-mediated responses greatly contribute to aging and senescence-associated vascular dysfunction in females. These early aging-related functional alterations in the vascular tree in females seem to appear earlier in conductance arteries than in resistance blood vessels, since they are observed in conductance, but not in resistance arteries (Jiménez-Altayó et al., 2013).

Aortic rings of male (6- to 7-month-old) SAMP8 mice also demonstrated reduced EDV (Lloréns et al., 2007), in agreement with other models (Delp et al., 1995). However, reduced vasodilation to acetylcholine in male SAMP-8 mice is not mediated by vasoconstrictor prostanoids or reduced eNOS expression, but is due to oxidative stress (Lloréns et al., 2007). In aortas of 12 month-old CD-1 male mice, treatment with indomethacin restored the impaired EDV, suggesting an involvement of increased COX-derived vasoconstrictors (Costa et al., 2016). In 12 month-old female CD1 mice, endothelial function is preserved by normal eNOS expression and an increased release of prostacyclin (Costa et al., 2016).

Endothelium-independent vasodilation is determined by measuring vascular responses to NO donors, as sodium nitroprusside, in mouse models and humans. In clinical studies, endothelium-independent vasodilation is preserved in both older men and premenopausal women in the micro (Gerhard et al., 1996; Desouza et al., 2000; Desouza et al., 2002) and macrocirculation (Taddei et al., 1996; Taddei et al., 2001). However, studies using doppler ultrasound showed that increases in blood flow induced by sodium nitroprusside in brachial and common femoral arteries are reduced in older men (Newcomer et al., 2004). Explanations for these discrepancies are the different blood flow measurement techniques and the wide age range among the older subjects included in the studies.

In animal models, such as 24-month-old male Fischer 344 rats, abdominal aorta, but not iliac and femoral arteries, exhibit decreased sodium nitroprusside-mediated relaxation (Luttrell et al., 2020). In 26–29 month-old male Wistar rats, sodium nitroprusside-induced relaxation was impaired in endothelium-intact, but not in endothelium-denuded aortic rings, suggesting an inhibitory influence of the endothelium on the response to this NO donor (Kim et al., 2011). In contrast, age had no effect on sodium nitroprusside-induced relaxation in thoracic aorta and femoral arteries of 33 month-old female Wistar rats (Barton et al., 1997) or in aortic rings of 30 month-old male Fischer 344 rats (Delp et al., 1995).

Therefore, there is still controversial information about a decreased sensitivity of vascular smooth muscle cells to NO or smooth muscle capacity of vasodilation in aging. Additional studies eliminating the interference of endothelium-derived factors on NO donor-mediated vasodilation in males and females are necessary. Although reduced NO bioavailability is well described in aged men and women, potential sex-dependent effects of antioxidants, or other agents aimed to rescue NO levels, may lead to new insights in this field.

### Reactive Oxygen Species

Oxidative stress, i.e., increased levels of ROS such as superoxide anion (O_2_^−^˙), hydrogen peroxide and hydroxyl radical, and reactive nitrogen species (RNS) such as peroxynitrite (Brandes et al., 2005; Costa et al., 2021), is directly linked to reduced NO bioavailability. The reaction between NO and O_2_^−^˙ produces the peroxynitrite radical, a cytotoxic compound that promotes DNA damage. The main sources of O_2_^−^˙ in blood vessels of older animals and humans are mitochondria (Ungvari et al., 2010; Tang et al., 2014), NADPH oxidase (Hamilton et al., 2001; Lassègue et al., 2012) and uncoupled eNOS (Delp et al., 2008; Li and Förstermann, 2013).

Oxidative stress and pro-inflammatory cytokines, such as tumor necrosis factor-α (TNF-α) contribute to endothelial dysfunction and large artery stiffening in postmenopausal women. Decreased NO bioavailability is considered a key factor contributing to the impaired endothelium dependent vasodilation in aging. Older adults present higher serum levels of inflammatory cytokines, such as interleukin-6 (IL-6), interleukin-1 (IL-1), TNF-α, and interleukin-18 (IL-18) when compared to younger adults (Wei et al., 1992; Hager et al., 1994). In addition, serum TNF-α concentrations are increased only in older healthy women (Straub et al., 1998).

Older men, 55–78 years, compared to young men (18–30 years), exhibit higher levels of nitrotyrosine, a marker of oxidative stress, in endothelial cells from the brachial artery and antecubital veins. No differences between the groups were detected for xanthine oxidase, antioxidant cytosolic (Cu/Zn-SOD) and mitochondrial (Mn-SOD) superoxide dismutase, or catalase (Van Der Loo et al., 2000). Also, older men exhibit elevated nicotinamide adenine dinucleotide phosphate (NAD(P)H) oxidase subunit p47phox in venous-derived endothelial cells compared with young subjects (Van Der Loo et al., 2000), and administration of vitamin C restores EDV in older healthy men (Eskurza et al., 2004; Jablonski et al., 2007). Studies with rodents also showed enhanced vascular NAD(P)H oxidase-derived O_2_^−^˙ production with unchanged xanthine oxidase-derived O_2_^−^˙ in arteries from old (18–20 months) compared with young (3–4 months) male Sprague-Dawley rats (Csiszar et al., 2002).

Increased ROS and imbalance of the antioxidants enzymes are a ubiquitous problem in vascular aging in men and women, with greater oxidative stress in men compared to premenopausal women (Ide et al., 2002). It is undeniable that the senior population is more susceptible to oxidative stress conditions, but further studies are needed to understand whether oxidative stress is an intrinsic aging process or, as we get older, we become more susceptible to oxidative injury.

ROS are fundamental to many endothelial cell functions, acting e.g. as signaling molecules that activate proinflammatory processes (Ungvari et al., 2007). ROS activates Nuclear Factor Kappa B (NF-κB) that regulates endothelial activation and expression of proinflammatory mediators, leading to endothelial senescence (Keymel et al., 2008). ROS signal the presence of a defective cell and stimulate progenitor cells to continue to replenish the circulatory system with functional differentiated endothelial and smooth muscle cells. Generally, senescent cells are removed by apoptosis and immune cells. Endothelial senescence should abrogate the process, but the immune system is ineffective in removing these endothelial cells. The extent of immune-system involvement in regulating age-related accumulation of senescent cells, and its consequences, are unknown. However, impaired cell cytotoxicity and defective function of circulating endothelial pluri-potent cells have been reported (Rauscher et al., 2003; Keymel et al., 2008). These defective endothelial cells can further promote senescent-cell accumulation and shorten lifespan, generating a chronic proinflammatory environment where cells continue to signal the immune system (Morgan et al., 2013).

Characterizations of peripheral blood mononuclear cells using ATAC-seq, RNA-seq, and flow cytometry showed less naïve T cells and increasing monocyte and cytotoxic cell functions in older patients (Márquez et al., 2020). These changes are greater in magnitude in men, and premature immunosenescence (Fuente et al., 2004) is accompanied by a male-specific decline in B-cell specific loci. These results are elegantly discussed by Márquez et al. (2020), and this field should be explored in mouse models of aging, since overactivation of the immune system increases the risk for atherosclerosis in men and pre/post-menopause women.

### Vasoconstriction

Increased vascular constrictor responses in aging may vary depending on the vasoconstrictor agent and vascular bed studied. In general, contractile responses to adrenergic vasoconstrictors and angiotensin-II (Ang II) are increased by chronological aging in female and male mice. Potential mechanisms include decreased endothelial NO synthase expression, reduced NO synthesis, increased ROS production, decreased SOD activity (Bolzán et al., 1997; Inal et al., 2001; Mariani et al., 2006); and increased vasoconstrictor prostanoids, especially in women (Li et al., 2008).

Aging reduces vasoconstriction to potassium chloride (KCl) in 24 month-old male Fischer rats, compared to 4 month-old rats. Removal of the endothelium increases KCl constrictor responses, but the age-associated decrease in KCl responsiveness remains (Shipley and Mullerdelp, 2005). Aortic rings of male (6- to 7-month-old) SAMP8 mice display greater contractility to KCl and phenylephrine (Lloréns et al., 2007), which is associated with a decreased modulatory effect of NO, but no alterations in smooth muscle cells function or structure. Angiotensin II-induced vascular contraction increases with aging in male and female CD-1 mice, but is higher in male mice (Costa et al., 2016). Angiotensin II is a key regulator of cell senescence, and modulates the onset and progression of vascular aging (Min et al., 2009; Wang et al., 2010). Increased Ang II responses in aorta of males CD1 mice contribute to endothelial dysfunction by a mechanism that partially involves Ang II-mediated upregulation of COX-derived vasoconstrictors (Costa et al., 2016). Reinforcing these findings, chronic angiotensin converting enzyme (ACE) inhibition, as well as Ang II type 1 (AT1) receptor blockade, recover endothelium-derived hyperpolarizing factor-mediated responses in mesenteric arteries of 12 month-old male Wistar-Kyoto rats (Goto et al., 2004).

It is not clear how prostaglandins are differently produced in aged males and females (Costa et al., 2016). In carotid arteries of 6-month-old female SAMP8 mice, increased phenylephrine vasoconstriction is related to increased Thromboxane (TXA_2_) production. Costa et al. (Costa et al., 2019) showed a differential interaction between estrogens and prostanoids production in menopause. While selective COX-1 or COX-2 inhibitors do not change phenylephrine responses in ovariectomized 6 month-old female SAMP8 mice that underwent an early-treatment with estrogen, COX inhibition decreases vasoconstrictor responses to phenylephrine in mice that receive a late estrogen-treatment. Further studies showed that aortas of female SAMP8 mice show lower NO levels, and consequently, less NO modulatory effects, in response to TXA_2_ receptor activation (Novella et al., 2013a). Indeed, prostanoids have a important participation in enhanced constriction in carotid arteries, but not in resistance mesenteric arteries from female SAMP8 mice (Jiménez-Altayó et al., 2013; Costa et al., 2019).

Functional vascular alterations appear earlier than structural changes with aging in male SAMP8 mice. Similarly, flow-mediated dilation is preserved in men aged < or = 40 years and declines thereafter at 0.21%/year, but remains preserved in women until the age of 50 years (Celermajer et al., 1994; Skaug et al., 2013). This could be an important information for targeting early signs of CVD in men and women, since coronary artery calcification (Howard et al., 1993), carotid intima-media thickening (Mcclelland et al., 2006) and atherosclerotic plaques are all more common in men than in women (pre-menopause) at young adulthood and middle-age (Kelley et al., 2011).

### Perivascular Adipose Tissue

The perivascular adipose tissue (PVAT) is no longer considered an inert vessel-supporting connective tissue, but an important regulator of vascular tone, exhibiting an anti-contractile effect in response to a variety of vasoconstrictors. This beneficial vasodilatory response is associated with anti-inflammatory effects of PVAT in animal models (Löhn et al., 2002; Dubrovska et al., 2003; Gollasch and Dubrovska, 2004; Verlohren et al., 2004) and in healthy individuals (Greenstein et al., 2009). Alterations in PVAT function may contribute to aging-associated vascular dysfunction and increased cardiovascular risk. However, few research groups have addressed aging-associated alterations in the PVAT, and, so far, the studies performed were carried out only in male animals.

Increased O_2_^−^˙ signaling (Fleenor et al., 2014) and increased advanced glycation end-products (AGE) accumulation (Ouyang et al., 2017) were reported in thoracic aortic PVAT of 27–29 month-old C57BL/6 male mice. In mesenteric arteries of 12 month-old SAMP8 male mice, PVAT anti-contractile effect to noradrenaline is absent in comparison with SAMR1, but mechanisms were not investigated (Agabiti-rosei et al., 2017). SAMP-8 mice at the age 12 months are close to the end of their lives and may already be riddled with aging-associated multiple disease injuries. These data reinforce the importance of mechanistic and descriptive studies of age-associated changes in PVAT in males and females.

### Other Potential Mechanisms

Considering that hormones that control reproduction modulate mitogenesis and differentiation, the Reproductive-Cell Cycle Theory of Aging states that reproductive hormones negatively regulate aging by promoting growth and development (Atwood et al., 2003; Bowen and Atwood, 2004). According to the theory, the period of maximum reproductive function is linked to the slowest aging period; and as reproductive function begins to decrease, typically during the fourth decade of life, the rate of change in body composition and function, and therefore aging, increases. Here we explore the mechanisms mediated by growth hormone (GH), insulin-like growth factor-1 (IGF1) and reproductive hormones axis on vascular aging.

#### Growth Hormone and Insulin-Like Growth Factor 1

In aging, secretion of GH, also known as somatotropin, and IGF-1 declines over time, with lowest levels in individuals aged ≥60 years, a phenomenon known as “somatopause” (Junnila et al., 2013). Increased IGF-1 signaling is associated with a decrease in longevity, and inhibition of mTOR by decreased GH/IGF-1 signaling stimulates autophagy, improving response to cellular stress, and increasing lifespan (Wullschleger et al., 2006; Fontana et al., 2010; Fulda et al., 2010).

Mutations in the IGF-1R gene that reduce IGF-1 signaling have been identified in centenarians (Suh et al., 2008), but men and women with deficiency of GH, thyroid-stimulating hormone (TSH), prolactin, follicle-stimulating hormone (FSH) or luteinizing hormone (LH) have normal longevity (Krzisnik et al., 1999; Besson et al., 2003; Krzisnik et al., 2010); some even reduced mean lifespan (Salvatori et al., 1999; Aguiar-Oliveira et al., 2010).

The cardiovascular system is an important target organ for GH and IGF-1. There is evidence that cardiac myocytes, vascular endothelial and smooth muscle cells abundantly express IGF1R and that they are more sensitive to IGF-1 than to insulin (Chisalita and Arnqvist, 2004). IGF-1 is important to maintain the functional and structural integrity of the microcirculation. IGF-1 increases NO bioavailability, decreased ROS generation, and has anti-inflammatory, antiapoptotic, and proangiogenic effects (Ungvari and Csiszar, 2012).

Recombinant human GH has been widely used to promote antiaging effects and cardiovascular protection although its efficacy has not been established (Rudman et al., 1990; van den Beld et al., 2003; Denti et al., 2004). Men and women with GH deficiency, who exhibit a pathological and often abrupt decline of GH secretion and, consequently, low levels for their age, show reduced flow-mediated EDV (M Evans et al., 1999), which is restored by GH replacement therapy (Smith et al., 2002). In addition, GH treatment of old individuals with no GH deficiency may increase the risk of other medical conditions (Blackman et al., 2002; Liu et al., 2007).

IGF-1 has significant proangiogenic effects in the heart and brain, inducing proliferation of microvascular endothelial cells through hypoxia-inducible factor 1-alpha (HIF-1α) and vascular endothelial growth factor (VEGF), i.e., *via* the canonical angiogenic pathway (Lopez-Lopez et al., 2004). IGF-1 also prevents oxidative distress by preserving the mitochondrial functional integrity (Li et al., 2009). These effects of IGF-1 on age-dependent impairment of angiogenesis are being explored in ischemia and aging neurodegeneration models (Rivard et al., 1999).

Studies linking IGF-1 levels to cardiovascular disease in elderly were inconclusive (Malozowski, 2003; Maggio et al., 2006). In aging, IGF-1 has been shown to recruit cardiomyoblasts, compensating for cell death and preventing ventricular dysfunction (Torella et al., 2004). GH supplementation, which increases circulating levels of IGF-1, increases cortical vascular density (Sonntag et al., 1997) and improves cognitive function in 24-month-old male Brown Norway×Fisher 344 rats (Ramsey et al., 2004; Sonntag et al., 2005). Treatment of 24-month-old Sprague-Dawley male rats with IGF-1 upregulates eNOS and improves bioavailability of NO in cavernosal arteries (Pu et al., 2008). In addition, age-dependent impairment of endothelial progenitor cells was abrogated by the GH-mediated increase in circulating IGF-1 in sixteen healthy middle-aged male volunteers (mean age 57.4 ± 1.4 years) and in aged (6–8 months old) male mice (Thum et al., 2007). Although acute GH-mediated increase in circulating IGF-1 seems to exert beneficial effects on the regenerative capacity of the cardiovascular system in elderly men. Data in women volunteers and female models are still needed.

Divergent data support that chronic modulation of IGF-1 promotes arterial obstructive lesions (Bayes-Genis et al., 2000; Higashi et al., 2014), since it induces vascular smooth muscle cell (VSMC) proliferation *in vitro* (Pfeifle et al., 1987) and increased IGF-1 and IGF-1 receptor have been shown in human and rabbit atherosclerotic arteries (Grant et al., 1996; Grant et al., 1999) compared with normal tissues (Grant et al., 1999). Future studies should elucidate whether age-related IGF-1 deficiency is further exacerbated by age-related changes in vascular expression of IGF-1–binding proteins (BPs), IGF-1 receptors or alterations in signaling pathways activated by IGF-1 receptors and whether it increases atherosclerosis risk in men and women.

#### Sex Hormones in Vascular Aging

The lower incidence of CVD in premenopausal women, relative to age-matched men, suggests a significant role for female gonadal hormones in the regulation of the vasculature (Mendelsohn and Karas, 2005). Aging *per se* decreases acetylcholine-induced relaxation, which is further reduced by the removal of ovaries. Accordingly, aging and hormonal status are associated with decreased endothelium-dependent and NO-mediated vasodilation (Taddei et al., 1996; Virdis et al., 2000; Teede, 2007). Aged female and male SAMP8 mice reproduce all the morphological (Goyal, 1982), mechanical (Reddy et al., 2003) and endothelial alterations (Blackwell et al., 2004) of the aged human aorta. However, aging-related hormonal changes are not quite similar in mice and humans.

The decline in ovarian function in aging women is usually mimicked by OVX procedures in mouse and rat research models. OVX is often performed due to the lack of natural menopause in rodents in an attempt to reproduce the human hormonal changes. However, most studies have used ovariectomized female rats at young ages (6–12 weeks old) to examine the effects of hormone deprivation in the cardiovascular system. This can lead to unreliable results given that the cardiovascular system has not yet “aged” (Wong et al., 2006).

Testosterone decreases markedly in male SAMP8 mice between 4 and 12 months of age, but the decrease in SAMR1 mice over the same period is not significant, suggesting that SAMP8 gonadal function parallels the decline in cognitive ability (Flood et al., 1995). On the other hand, 6-month-old female SAMP8 mice exhibit hormonal status similar to SAMR1 (Novella et al., 2010; Novella et al., 2013a; Novella et al., 2013b). Thus, SAMP8 mice might represent a valuable model to study the mechanisms of vascular aging at a convenient standard time course (Novella et al., 2010; Novella et al., 2013a) and without overlapping the deleterious effects of estrogen reduction. Of importance, the lack of estrogen protection in SAMP8 mice is not related to age-associated changes in the plasma levels of estrogen or activity of estrogen receptors, but rather to potential age-related changes in estrogen mediated signaling pathways in the vasculature.

Estrogen levels affect arterial distensibility (Gangar et al., 1991). However, there are also non-hormonal differences that affect the behavior of the arterial tree. In men, it is unclear whether endothelial dysfunction occurs with age-associated declines in testosterone in the absence of disease. Cross-sectional association studies aiming at serum testosterone levels and endothelial function have shown that low serum testosterone is associated with reduced (average age 52.8 years) (Akishita et al., 2007; Empen et al., 2012) and increased (average age 55.9 years) (Mäkinen et al., 2011) macro- and microvascular endothelial function in men. One reason for these divergent results could be that testosterone concentrations exhibit significant diurnal and day-to-day variations (Bhasin et al., 2018). Another drawback is that the measurement of total testosterone, commonly performed *via* direct assay (radioimmunoassay, enzyme-linked immunosorbent assay, or chemiluminescent immunoassay) has limited accuracy, especially in lower testosterone ranges (<300 ng/dl), with testosterone concentrations being frequently overestimated (Rosner et al., 2007). Also, measurements of total testosterone include the fraction that is tightly bound to sex hormone-binding globulin (SHBG), which increases with age (Harman et al., 2001) and might be a bias for men with conditions that affect SHBG like obesity, or type 2 diabetes mellitus.

This variation in testosterone measurement is reflected in the clinical setting, and occurrence of low testosterone without symptoms does not meet the definition of “androgen deficiency” set by the Endocrine Society (Bhasin et al., 2018). Although testosterone declines by approximately 1% per year in men after the third decade (Matsumoto, 2002), decay of bioavailable testosterone is even greater than decay in total testosterone. Consequently, male aging studies should look at modest increases in FSH and LH, impairments in testis function and hypothalamic regulation of gonadotropin secretion that accompanies testosterone decline (Matsumoto, 2002).

#### Hormonal Receptors Signaling

Estrogen triggers NO release *via* estrogen receptor (ER)α-mediated activation of eNOS as well as increased eNOS transcription (Klinge, 2001). Few studies have shown whether aging in female rodents is associated with significant reduction of estrogen-mediated cardiovascular effects. Aging influences the activation of receptors (Novella et al., 2010; Mehta et al., 2019). The discovery of age-dependent decline of receptor function emerged in several laboratories during the last decades of the 20th century.

This aspect, the loss of receptors function, is, however, still ignored by physicians, even by geriatricians, in the prescription of drugs and hormones to senior people (Robert and Fulop, 2014). Dr. George Roth research group at the National Institute of Aging (NIA) first showed the aging-dependent decline in muscarinic receptor responsiveness (Joseph et al., 1990). Of importance, aging modulation of receptor signaling seems to occur in both sexes and affects the vascular system equally in men and women (Faury et al., 1997; Robert, 1998; Xiao et al., 1998; Novella et al., 2012b; Moreau et al., 2020).

Detailed analysis suggests that early initiation of estrogen therapy produces more favorable results than the average late-onset, which is used in most clinical trials (Grodstein et al., 2006). The so-called “timing hypothesis” (Novella et al., 2010; Novella et al., 2012b) relies on the concept that estrogen has beneficial effects if taken before, or close to, the onset of menopause. Although aging-dependent detrimental effects of estrogen in the vasculature have not yet been demonstrated (Harman et al., 2011), it is possible that aging can determine abnormal responses to therapy in older women, which would be linked to changes in the classical signaling of endogenous hormones.

A key part in maintaining correct cellular function and thus healthiness of the organism is proper gene expression and regulation. Alternative splicing of RNA transcripts, i.e., the formation of alternative splicing receptors, might alter or modulate receptor signaling. Functional experiments in common carotid artery of senescent SAMP8 female mice showed that late-onset of estrogen treatment increases adrenergic vasoconstriction along augmented TXA_2_ production and upregulation of ER36 expression, an alternative splicing of the classical estrogen receptors (ER) (Costa et al., 2019). Also, an age-related increase in methylation-associated inactivation of genes encoding ERs has been described, and ER methylation in atherosclerotic plaques is higher than in non-plaque regions in vascular tissues (Post et al., 1999; Kim et al., 2007).

Pre-mRNA splicing is an intricate post-transcriptional process that leads to the removal of introns and joining of exons in a pre-mRNA to form a mature mRNA (House and Lynch, 2008). A well-studied example is the Hutchinson Gilford progeria syndrome, where a silent point mutation in the lmna gene adds a 5’ splicing signal, leading to a shortened transcript and subsequently a shorter version of its encoded protein Lamina A9 (Liao and Kennedy, 2014). Patients with Hutchinson Gilford progeria syndrome suffer from extensive atherosclerosis and cardiac electrophysiological alterations that invariably lead to premature aging and death (Osorio et al., 2011). Since many components of the RNA processing machinery are themselves regulated by alternative splicing, defects in differential splicing of genes might, therefore, catalyze the aging process *via* a feed forward mechanism.

Global genome splicing analysis in young and old patients reveals an increased number of alternatively spliced genes related to skin and skeletal muscle (Rodríguez et al., 2016). Since sex hormones are important in cell signaling transcription, further studies are necessary to understand their roles in impaired RNA processing and translation. The role of miRNA and possible miRNA targets in elderly women are elegantly discussed in a review by Pérez-Cremades et al. (2018).

New studies suggest that age-dependent modifications of nuclear hormones may indeed play an important role in the age-dependent decline of several biological functions of vital importance. Studies in mouse models can help to understand the importance of age-dependent modifications of receptor function in the vascular system as these effects are accelerated in the SAMP8 mouse, an appropriate model to study vascular effects of aging. It’s important to fulfill the gaps on age-dependent endothelial dysfunction in large arteries of males and females and to determine whether this precedes the development of CVD. In the SAMP8 mice, vascular studies should be performed up to 6–7 in males and 8–10 months in females. After 9–10 months SAMP mice begin to develop other pathologies such as insulin resistance, hyperglycemia, hyperinsulinemia and high levels of free fatty acids (Cuesta et al., 2013; Liu et al., 2015).

## Clinical Perspective and Conclusions

Senescence and aging are two distant processes, but they are conceptually intertwined. Women and men grow old, but in different ways. The different mechanisms in vascular aging are associated in part with hormonal changes, inflammation and oxidative stress. Regardless of sex differences in the aging of the cardiovascular system, effective treatment of CVD in women is a challenging issue in medicine, mainly due to the lack of information on the mechanisms involved in the initial stage of CVD, symptoms, and menopause process.

The ability to identify individuals having early deterioration of vascular and cardiac function, as well as progressive subclinical arterial disease, would allow to define a target population for therapy that reduces vascular and cardiac remodeling and dysfunction, and prevents lethal or debilitating events. Considering clinical applications, organismal senescence at the organ level has already been classified in the International Classification of Disease code (ICD) as ‘‘intrinsic aging of the skin” and “photoaging of the skin,” (Zhavoronkov, 2020), but many diseases within the ICD do not consider ageing in their categorization. In addition, preventive treatment for what is now considered normal cardiovascular aging, may become part of a routine check-up - e.g., a test for endothelial dysfunction, a marker/predictor of vascular aging. Also, reference values should be different in men and women? Should men start vascular checkup at an earlier age than women? Should aging-related alterations be investigated for preventive treatment? Certainly, results from further studies will contribute to clarify these questions.

## Glossary

ACE: Angiotensin converting enzyme
Ang II: Angiotensin-II
AT1: Ang II type 1 receptor
BH4: Tetrahydrobiopterin
BLSA: The Baltimore Longitudinal Study on Aging
COX-1: Cyclooxygenase-1
COX-2: Cyclooxygenase-2
COX: Cyclooxygenase
Cu/Zn-SOD: Cytosolic superoxide dismutase
CVD: Cardiovascular diseases
EDV: Endothelium-dependent vasodilation
ER: Estrogen receptor
ERE: Estrogen-responsive element
FHS: The Framingham cohort study
FSH: Follicle-stimulating hormone
GH: Growth Hormone
HIF-1α: Hypoxia-inducible factor 1-alpha
hTERT: Telomerase reverse transcriptase
ICD: International Classification of Disease Code
IGF-1: Insulin-like Growth Factor 1
IL-1: Interleukin-1
IL-18: Interleukin-18
IL-6: Interleukin-6
KCl: Potassium chloride
L-NAME: L-NG-Nitroarginine Methyl Ester
LH: Luteinizing hormone
Mn-SOD: Mitochondrial superoxide dismutase
NAD(P)H: Nicotinamide adenine dinucleotide phosphate
NF-κB: Nuclear factor kappa B
NIA: National Institute of Aging
NO: Nitric oxide
O_2_^−^•: Superoxide anion
OVX: Ovariectomy
PVAT: Perivascular adipose tissue
RNS: Reactive nitrogen species
ROS: Reactive oxygen species
SAMP: Senescence-accelerated mouse prone
SAMR: Senescence-accelerated resistant mouse prone
SASP: Senescence-associated secretory phenotype
SHBG: Sex hormone-binding globulin
SOD: Superoxide dismutase
TNF-α: Tumor necrosis factor-α
VEGF: Vascular endothelial growth factor
VSMC: Vascular smooth muscle cell
